# Marginal ancestral contributions to atrial fibrillation in the Standardbred racehorse: Comparison of cases and controls

**DOI:** 10.1371/journal.pone.0197137

**Published:** 2018-05-15

**Authors:** Megan Kraus, Peter Physick-Sheard, Luiz F. Brito, Mehdi Sargolzaei, Flávio S. Schenkel

**Affiliations:** 1 Department of Animal Biosciences, University of Guelph, Guelph, Ontario, Canada; 2 Department of Population Medicine, University of Guelph, Guelph, Ontario, Canada; 3 The Semex Alliance, Guelph, Ontario, Canada; University of Sydney Faculty of Veterinary Science, AUSTRALIA

## Abstract

Admissions of Standardbred racehorses (**Std**) to the Ontario Veterinary College Teaching Hospital (**OVCTH**) for treatment of atrial fibrillation (**AF**) began to increase in the early 1990s. The arrhythmia has been shown to have a modest heritability (h^2^ ≃ 0.15), with some stallions appearing as sires or sires of mares used in breeding (broodmares) of affected horses more frequently than others. The objective of this study was to determine the marginal genetic contributions of ancestors to cohorts of Std affected with AF and their contemporary control groups, and whether these ancestors contribute significantly more to the affected cohorts than to controls. All Std admitted to OVCTH for treatment of AF that were born between 1993 and 2007 comprised the affected case group (n = 168). Five randomly selected racing contemporaries for each Std admitted, assumed to not suffer from the arrhythmia, comprised the control group (n = 840). Three-year overlapping cohorts were created for case and control horses, determined according to year of birth, for a total of 26 cohorts. Marginal genetic contributions of ancestors to each cohort were determined and differences analyzed for statistical significance using a two-tailed paired t-test, with P ≤ 0.05 considered significant. The marginal contributions of 26 ancestors were significant, with 11 contributing significantly more to affected cohorts than the corresponding controls, and 15 contributing significantly more to controls than the corresponding affected cohorts. One stallion and one broodmare were very highly significant to affected cohorts at P ≤ 0.001, and nine stallions and three broodmares were very highly significant to control cohorts at P ≤ 0.001. Therefore, a number of stallions have statistically significant contributions to the genetics of Std affected with AF, while many others have statistically significant contributions to healthy Std. The arrhythmia appears to be particularly prevalent in the descendants of one sire family.

## Introduction

Atrial fibrillation (**AF**) is the most common clinically significant arrhythmia in horses, and has a major impact on high-performance equine athletes [1, 2]. It appears most prevalent in racehorses, particularly the Standardbred racehorse (**Std**) [3–5]. With the loss in training and competition time, potential for a sustained decrease in performance ability, and the expense and manner of treatment, it is reasonable to assume that there is concern from both an economic and welfare standpoint when considering AF in the horse.

Evidence points towards there being a genetic background to the arrhythmia in the Std [6–8]. Most instances of familial AF in humans affect younger individuals and have an autosomal dominant mode of inheritance. This is encouraging for potential research into the genetics of AF in the horse, as it is known that in horses AF shows no clear relationship with age, so young racehorses are susceptible to the arrhythmia [9], and many of the known genetic diseases of the horse are also inherited in an autosomal recessive or dominant manner [10]. Because AF has both economic and welfare implications, efforts should be made to decrease the number of Std with a potential to develop the disease. Two previous publications describe analysis of this dataset [7, 8]. In the first paper, breed incidence of AF was described, heritability and inbreeding estimates were determined, and the frequency of individual sires (1^st^ generation back in the pedigree) in case and control groups were compared. The second paper extended these analyses and examined AF, stratifying by gait and gender. These papers established the basis for genetic liability to the condition in the breed and showed potential differences between gaits and genders with possibly larger heritability estimates in males and pacers. The present study extends the analysis further, examining marginal ancestral contributions.

One way to improve further our understanding of the genetic basis of AF would be to determine the ancestral genetic contributions to the genetic pool of the group of horses that appear to be more susceptible to the arrhythmia compared to those of the group of control, unaffected horses. Analysis of a population for probabilities of gene origin allows the determination of ancestral marginal contributions [11]. This type of analysis can discriminate horses or families more associated with the incidence of AF, allowing for better understanding of its genetic background. Therefore, the objectives of this study were to determine the marginal genetic contributions of ancestors to affected and control Standardbred racehorses, and to identify those highly contributing ancestors that have significantly larger contributions to affected horses than to controls.

## Material and methods

The authors have confirmed that no Animal Care Committee approval was necessary for this particular study, as all information required was obtained from existing databases. All clinical cases were treated prior to this study. Owner-informed consent to the use of the dataset was obtained for use of the breed registry dataset via the breeder association.

### Selection of case and control groups

The group of affected animals (**A**) and their contemporary control group (**C**) is a subset of the group described previously by Kraus et al. [7]. Admissions of Standardbreds with AF to the Ontario Veterinary College Teaching Hospital (**OVCTH**) began to markedly increase starting in 1993, thus the pedigrees of all case and control horses born between 1993 and 2007, inclusive, were examined. Stratifications by sex and gait were not used in order to avoid problems stemming from small sample sizes. Ancestral contributions were determined for overlapping birth-year cohorts, three years at a time, allowing for the change in marginal contributions to be followed closely over time. The first cohorts evaluated were for the birth years 1993, 1994, and 1995 (affected: A93/95, control: C93/95). Subsequent analyses looked at birth years of 1994, 1995, and 1996, (A94/96, C94/96), etc., up to and including 2007 (A05/07, C05/07). For each cohort, the pedigrees were traced to the earliest known ancestors.

### Descriptive statistics of pedigrees

In order to obtain an understanding of the complexity and depth of the pedigrees of affected and control Std, descriptive details were obtained through CFC software [12], including average levels of inbreeding, five-generation pedigree completeness index (PCI) [13], number of sires, number of dams, and average genetic relationship for Std within each cohort.

### Marginal genetic contributions

The objective of this study was to determine if contributions of ancestors to the case group differed significantly from contributions of those same ancestors to the control group, therefore the marginal genetic contributions to 13 control cohorts were measured, one for each affected case cohort [11]. Ancestors were chosen based on their genetic contribution and are, therefore, not necessarily founders of the population. The highest-contributing ancestors were chosen one by one in an iterative process, starting with the highest-contributing ancestor, and proceeding to the lowest-contributing ancestor, with pedigree information updated for each iteration. This process is explained in detail by Boichard *et al*. [11]. An ancestor’s marginal contribution, p_k_, is defined as that contribution which has not yet already been explained by the *n-1* previously accounted-for ancestors. When calculating these marginal contributions, it is necessary to remove the possibility of the contributions of an ancestor being counted more than once. For the first method of doing so, the *n-1* selected ancestors may be ancestors of an individual, *k*, and therefore the genetic contributions, *a*_*i*_ of these *n-1* individual ancestors are adjusted for according to the equation: *p*_*k*_ *= q*_*k*_ * (1 - Σa_*i*_*)*, so that the marginal contribution of an ancestor, *p*_*k*_, does not include the genetic contributions already explained by the *n-1* ancestors. Additionally, it is possible that some of the previous *n-1* ancestors may be descendants of individual *k*, therefore, after each major ancestor has been found, and their raw and marginal contributions determined, the information on their sire and dam is deleted, making them a “pseudo-founder,” and avoiding the possibility of their contributions being attributed to ancestor *k*. The *prob_orig* function of the Pedig software [14] was used to complete these iterative calculations.

The number of ancestors explaining a certain % of the genetic pool (i.e., genetic variability) was computed from the marginal contributions of the ancestors given by the Pedig software [14]. This is possible, because the marginal distributions are orthogonal, i.e. there is no double counting of contributions, starting from the most contributing ancestor further back in the pedigree.

In order to identify the most important ancestors according to the magnitude of their marginal genetic contributions, the marginal genetic contributions of ancestors to each of all 13 affected or control cohorts were averaged. Ancestors with an average non-zero contribution of 0.5% or greater, that contributed to two or more non-overlapping cohorts, were identified. The contributions of the highest-contributing ancestors identified in this manner were analyzed using a paired two-tailed t-test, comparing their genetic contributions over all 13 three-year cohorts of affected Std to their contributions to the corresponding control cohorts. In all, 41 ancestors were tested for significance. A false discovery rate (FDR) [15] of 5% was used to control for multiple hypotheses test.

## Results

The number of individuals in each cohort, as well as the complete pedigree size, including the animals in each cohort, are shown in Table 1. Marginal ancestral contributions were determined, and the most influential ancestors to the affected and control cohorts were examined for statistical significance through a pairwise two-tailed t-test comparing their contributions to each affected cohort versus the corresponding control cohort.

**Table 1:** Number of individuals per cohort, and size of the complete pedigree for each three-year cohort of affected (A) or control (C) racehorses used to determine the marginal ancestral contributions to each.

Cohort	Size of cohort	Size of pedigree	Cohort	Size of cohort	Size of pedigree
**A93/95**	32	1,783	**C93/95**	160	3,887
**A94/96**	37	1,873	**C94/96**	185	4,072
**A95/97**	41	2,011	**C95/97**	205	4,389
**A96/98**	48	2,081	**C96/98**	240	4,912
**A97/99**	38	1,881	**C97/99**	190	4,403
**A98/00**	32	1,707	**C98/00**	160	4,210
**A99/01**	24	1,561	**C99/01**	120	3,560
**A00/02**	35	1,836	**C00/02**	175	4,285
**A01/03**	46	2,046	**C01/03**	230	4,821
**A02/04**	45	1,980	**C02/04**	225	4,861
**A03/05**	39	1,830	**C03/05**	195	4,463
**A04/06**	27	1,695	**C04/06**	135	4,019
**A05/07**	19	1,633	**C05/07**	95	3,444

### Descriptive statistics of pedigrees

As seen in Table 2, while not large, there was an overall trend for increase in inbreeding over time in both affected and control groups, but no clear pattern exists for any differences in average inbreeding coefficient between affected and control Std. Affected Std are seen to have a greater degree of relatedness than control Std, and cohorts of both groups have a high degree of pedigree completeness within five generations. Using the same Std as part of a larger data-set, Physick-Sheard *et al*. [8] reported no effect of inbreeding coefficient on the liability to AF (P = 0.91). These authors also reported that there was a significant difference in the distribution of inbreeding coefficients of affected and control Std, with control Std falling more in higher inbreeding classes than affected horses, indicating no effect of inbreeding on incidence of the arrhythmia.

**Table 2:** Descriptive pedigree statistics for each affected and control cohort showing average, maximum, and minimum inbreeding, number of sires, number of dams, average genetic relationship, and average 5-generation pedigree completeness index (PCI).

Cohort	Average inbreeding coefficient	Maximum inbreeding coefficient	Minimum inbreeding coefficient	Number of sires	Number of dams	Average relationship	Average PCI
**A93/95**	0.0824	0.1153	0.0394	23	32	0.1841	0.9994
**C93/95**	0.0820	0.1882	0.0300	129	159	0.1466	0.9963
**A94/96**	0.0834	0.1302	0.0394	23	37	0.1869	0.9992
**C94/96**	0.0849	0.1882	0.0300	140	183	0.1463	0.9962
**A95/97**	0.0837	0.1302	0.0394	28	41	0.1784	0.9988
**C95/97**	0.0848	0.1882	0.0310	154	204	0.1452	0.9967
**A96/98**	0.0875	0.1421	0.0550	28	47	0.1811	0.9985
**C96/98**	0.0857	0.1646	0.0310	178	238	0.1439	0.9971
**A97/99**	0.0851	0.1421	0.0496	23	38	0.1870	0.9985
**C97/99**	0.0848	0.1642	0.0368	149	187	0.1445	0.9978
**A98/00**	0.0875	0.1421	0.0496	18	32	0.2027	0.9988
**C98/00**	0.0867	0.1645	0.0368	130	158	0.1455	0.9982
**A99/01**	0.0838	0.1411	0.0440	16	24	0.2251	0.9997
**C99/01**	0.0896	0.1645	0.0448	105	118	0.1518	0.9986
**A00/02**	0.0908	0.1501	0.0440	23	35	0.2212	1.0000
**C00/02**	0.0907	0.1712	0.0325	132	175	0.1522	0.9978
**A01/03**	0.0898	0.1501	0.0440	23	46	0.2276	0.9997
**C01/03**	0.0930	0.1831	0.0325	161	230	0.1530	0.9984
**A02/04**	0.0907	0.1501	0.0610	18	45	0.2473	0.9997
**C02/04**	0.0947	0.1831	0.0325	159	223	0.1530	0.9984
**A03/05**	0.0917	0.1488	0.0610	15	39	0.2529	0.9997
**C03/05**	0.0965	0.1831	0.0375	145	194	0.1550	0.9996
**A04/06**	0.0994	0.1598	0.0680	15	27	0.2485	1.0000
**C04/06**	0.0990	0.1639	0.0505	112	134	0.1565	0.9996
**A05/07**	0.1022	0.1598	0.0680	13	19	0.2547	1.0000
**C05/07**	0.0987	0.1639	0.0505	82	94	0.1594	0.9997

### Marginal genetic contributions

**Fig 1:**
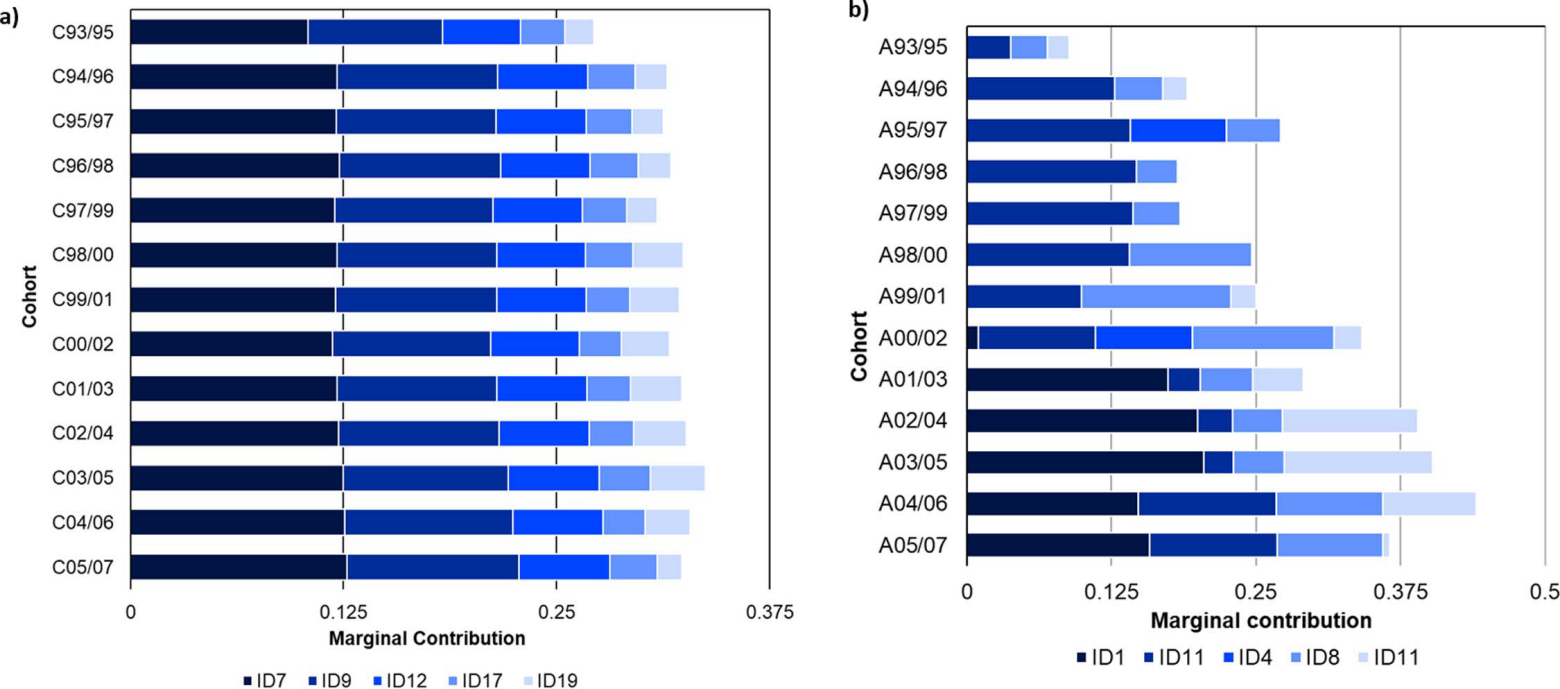
Marginal genetic contributions of the five highest-contributing ancestors significant to control (Fig 1A) and affected (Fig 1B) cohorts, selected according to largest average non-zero contribution.

Marginal contributions of individual ancestors to the control cohorts remained fairly consistent over time (Table 3). The highest contribution by any single ancestor (ID7), ranged from 11.9% to C00/02, to 12.7% to C05/07. This ancestor was the highest contributor to all control cohorts except for cohort C93/95, when it ranked second to ID11. The 10 highest contributing ancestors to the second control cohort, C94/96, maintained their high level of contribution to all subsequent cohorts with some slight alterations in order. The consistency of ancestral contributions to control cohorts is depicted in Fig 1A, which shows the contributions of the five highest-contributing ancestors to control cohorts.

**Table 3:** Marginal genetic contributions to three-year cohorts of control horses from 41 highest-contributing ancestors (Anc.) and overall average contribution (Ave.).

Anc.	93/95	94/96	95/97	96/98	97/99	98/00	99/01	00/02	01/03	02/04	03/05	04/06	05/07	Ave.*
**ID1**	-	-	-	-	-	-	-	-	-	-	-	-	-	-
**ID11**	0.125	0.036	0.035	0.032	0.032	0.032	0.032	0.034	0.031	0.031	0.026	0.029	0.023	0.038
**ID14**	0.086	-	-	-	-	-	-	-	-	-	-	-	-	0.086
**ID4**	-	-	-	-	-	-	-	-	-	-	-	-	-	-
**ID21**	0.035	0.108	0.110	0.104	0.108	0.105	0.111	0.111	0.107	0.104	0.100	0.097	0.093	0.099
**ID6**	0.075	0.087	0.088	0.087	0.087	0.085	0.085	0.085	0.086	0.085	0.086	0.087	0.088	0.085
**ID7**	0.104	0.121	0.121	0.122	0.120	0.121	0.120	0.119	0.121	0.122	0.125	0.126	0.127	0.121
**ID8**	0.014	0.013	0.011	0.011	0.011	0.025	0.030	0.043	0.043	0.042	0.041	0.041	0.045	0.028
**ID9**	0.079	0.094	0.094	0.095	0.093	0.094	0.095	0.093	0.094	0.094	0.097	0.099	0.101	0.094
**ID10**	0.062	0.075	0.075	0.074	0.074	0.073	0.071	0.073	0.070	0.069	0.068	0.065	0.064	0.070
**ID2**	-	-	-	-	-	0.003	-	-	-	-	-	-	-	0.003
**ID12**	0.046	0.053	0.053	0.053	0.052	0.052	0.052	0.052	0.053	0.053	0.053	0.053	0.053	0.052
**ID13**	0.001	0.034	0.034	0.033	0.035	0.033	0.035	0.031	0.030	0.030	0.029	0.027	0.026	0.029
**ID3**	-	-	-	-	-	-	-	-	-	-	-	-	-	-
**ID15**	-	-	0.013	0.021	0.027	0.024	-	-	0.017	0.020	0.018	0.019	-	0.020
**ID16**	0.008	0.004	0.006	0.006	0.008	0.003	0.012	0.012	0.014	0.014	0.015	0.013	0.012	0.010
**ID17**	0.026	0.028	0.027	0.028	0.026	0.028	0.026	0.025	0.026	0.026	0.030	0.025	0.028	0.027
**ID18**	0.028	0.030	0.030	0.031	0.029	0.013	0.013	0.013	0.013	0.013	0.013	0.012	0.028	0.020
**ID19**	0.017	0.019	0.018	0.019	0.018	0.029	0.029	0.028	0.030	0.031	0.033	0.026	0.014	0.024
**ID20**	0.022	0.020	0.020	0.019	0.019	0.016	0.019	0.019	0.020	0.019	0.020	0.019	0.017	0.019
**ID5**	-	-	-	-	-	-	-	-	-	-	-	-	-	-
**ID22**	-	-	-	-	-	0.009	-	-	-	-	-	-	-	0.009
**ID23**	0.010	0.012	0.014	0.016	0.014	0.012	0.012	0.012	0.014	0.014	0.015	0.016	0.020	0.014
**ID24**	-	-	-	-	-	-	0.006	-	-	-	-	-	-	0.006
**ID25**	-	-	-	-	-	-	-	-	-	-	-	-	-	-
**ID26**	-	-	-	-	-	-	-	-	-	-	-	-	-	-
**ID27**	0.007	0.007	0.007	0.007	0.007	0.007	0.007	0.006	0.006	0.006	0.006	-	0.006	0.007
**ID28**	0.013	0.016	0.017	0.019	0.018	0.020	0.018	0.018	0.021	0.023	0.025	0.025	0.023	0.020
**ID29**	0.018	0.017	0.017	0.014	0.014	0.013	0.011	0.011	0.011	0.011	0.011	0.010	0.013	0.013
**ID30**	0.013	0.015	0.010	0.012	0.011	0.019	0.020	0.019	0.020	0.021	0.023	0.034	0.036	0.019
**ID31**	0.006	0.007	0.007	0.007	0.006	0.007		0.007	0.006	0.006	0.005	0.005	0.004	0.006
**ID32**	-	-	-	-	-	-	-	-	-	-	-	-	-	-
**ID33**	-	-	-	-	-	-	-	-	-	-	-	-	-	-
**ID34**	-	-	-	-	-	-	-	-	-	-	-	-	-	-
**ID35**	-	-	-	-	-	-	-	-	-	-	-	-	-	-
**ID36**	0.002	0.002	0.002	0.002	0.002	0.001	0.001	0.001	0.001	0.001	0.001	0.002	0.001	0.001
**ID38**	0.011	0.010	0.003	0.004	0.005	0.004	0.020	0.018	0.005	0.004	-	0.006	-	0.008
**ID41**	0.007	0.008	0.008	0.008	0.008	0.007	0.007	0.007	0.008	0.008	0.008	0.009	0.009	0.008
**ID37**	0.007	0.008	0.008	0.008	0.008	0.007	0.007	0.007	0.007	0.007	0.008	0.008	0.008	0.007
**ID40**	0.007	0.008	0.008	0.008	0.008	0.007	0.007	0.007	0.007	0.007	0.007	0.007	0.007	0.007
**ID39**	0.004	0.005	0.005	0.006	0.005	0.005	0.005	0.005	0.005	0.004	0.005	0.006	0.006	0.005

*Ancestors with no values in all columns are ancestors that had marginal contributions to the affected group, but not to the control group.

Marginal contributions to the affected cohorts varied much more than those seen with the control cohorts (Table 4). The highest marginal contribution by a single ancestor, ID1, was 20.5% to A03/05. This ancestor was initially highlighted for interest as he has many offspring in the group of affected horses, and was the highest-ranked marginal contributor in cohorts A01/03 to A05/07. ID1 entered stud in 2001, therefore his first crop of foals were born in 2002. This is reflected in the first appearance of ID1 as a contributing ancestor to A00/02. The variation in levels of contributions of ancestors to affected cohorts can be seen in Fig 1B by the contributions of the five highest-contributing ancestors that were significant to affected cohorts.

**Table 4:** Marginal genetic contributions to three-year cohorts of affected horses from 41 highest-contributing ancestors and overall average contribution.

Anc.	93/95	94/96	95/97	96/98	97/99	98/00	99/01	00/02	01/03	02/04	03/05	04/06	05/07	Ave.
**ID1**	-	-	-	-	-	-	-	0.010	0.174	0.200	0.205	0.148	0.158	0.149
**ID11**	0.038	0.128	0.142	0.147	0.144	0.141	0.099	0.101	0.028	0.030	0.026	0.119	0.110	0.096
**ID14**	-	0.094	-	0.095	0.100	-	-	-	-	-	-	0.066	0.071	0.085
**ID4**	-	-	0.083	-	-	-	-	0.084	-	-	-	-	-	0.083
**ID21**	0.127	0.047	0.086	0.038	0.046	0.094	0.141	0.142	0.115	0.093	0.088	0.033	0.035	0.083
**ID6**	0.116	0.102	0.057	0.099	0.090	0.085	0.051	0.039	0.066	0.075	0.074	0.067	0.075	0.077
**ID7**	0.084	0.069	0.100	0.066	0.064	0.061	0.080	0.070	0.089	0.055	0.054	0.043	0.051	0.068
**ID8**	0.032	0.042	0.047	0.036	0.041	0.106	0.129	0.122	0.045	0.043	0.044	0.093	0.092	0.067
**ID9**	0.087	0.076	0.068	0.070	0.069	0.059	0.051	0.049	0.067	0.055	0.053	0.048	0.057	0.062
**ID10**	0.088	0.076	0.019	0.074	0.072	0.067	0.051	0.017	0.074	0.062	0.058	0.045	0.049	0.058
**ID2**	0.019	0.021	-	-	-	-	0.022	0.024	0.044	0.117	0.128	0.081	0.006	0.051
**ID12**	0.050	0.036	0.041	0.042	0.035	0.033	0.028	0.027	0.033	0.021	0.030	0.024	0.028	0.033
**ID13**	0.038	-	0.031	0.000	-	0.042	0.036	0.041	0.027	0.032	0.023	-	-	0.030
**ID3**	-	-	0.016	0.011	0.016	0.025	0.069	-	-	-	-	-	-	0.027
**ID15**	0.026	0.007	0.017	0.019	0.026	0.020	-	0.017	0.023	0.027	0.026	0.029	0.031	0.022
**ID16**	0.019	0.023	0.005	-	-	-	0.004	0.056	0.018	0.012	0.014	0.019	0.024	0.019
**ID17**	0.023	0.024	0.024	0.019	0.022	0.015	0.012	0.016	0.014	0.013	0.013	0.016	0.020	0.018
**ID18**	0.024	0.025	0.010	0.021	0.020	0.015	0.012	0.015	0.016	0.015	0.015	0.017	0.019	0.017
**ID19**	-	-	0.025	-	-	-	-	0.008	-	-	-	-	0.012	0.015
**ID20**	0.013	0.012	0.017	0.017	0.022	0.016	0.013	0.017	0.011	0.011	0.010	0.014	0.016	0.015
**ID5**	0.002	-	0.002	0.026	0.012	0.026	-	-	-	-	-	-	-	0.013
**ID22**	-	-	-	0.001	-	0.012	0.025	-	-	-	-	-	-	0.012
**ID23**	-	0.009	0.013	0.010	0.015	-	-	-	-	-	-	-	-	0.012
**ID24**	0.005	-	-	-	0.016	0.019	-	0.006	0.007	-	-	-	-	0.011
**ID25**	-	0.007	0.006	-	-	-	0.017	-	-	-	-	-	-	0.010
**ID26**	0.017	-	-	-	-	-	-	-	0.007	0.006	-	-	0.009	0.009
**ID27**	0.012	0.012	0.003	0.012	0.009	0.006	-	-	0.009	0.008	0.008	0.010	-	0.009
**ID28**	0.011	0.010	0.012	0.008	0.007	-	-	0.007	0.007	0.005	-	-	0.010	0.008
**ID29**	0.010	0.011	0.008	0.007	0.009	0.006	-	0.007	0.008	0.008	0.008	-	-	0.008
**ID30**	0.009	0.016	0.008	0.007	0.007	0.009	-	0.010	0.004	-	0.002	-	-	0.008
**ID31**	-	0.001	0.009	0.013	0.004	0.004	0.005	-	-	0.006	0.006	0.009	0.009	0.007
**ID32**	0.006	-	-	-	0.005	0.007	0.007	-	-	-	-	-	-	0.006
**ID33**	0.003	0.003	0.011	0.009	0.002	-	-	-	-	-	-	-	-	0.006
**ID34**	0.008	0.009	0.001	-	-	-	-	0.008	-	-	0.002	-	-	0.006
**ID35**	0.002	0.002	-	0.007	0.010	0.010	0.009	0.005	0.003	0.002	-	-	-	0.005
**ID36**	0.007	0.006	-	0.006	0.005	-	-	-	0.005	0.005	0.003	-	-	0.005
**ID37**	0.005	-	0.005	0.005	0.004	-	-	0.004	0.005	0.005	-	-	-	0.005
**ID38**	-	-	0.001	0.002	0.002	0.002	0.013	-	-	-	-	-	-	0.004
**ID39**	-	-	0.003	-	-	-	-	-	-	-	-	-	-	0.003
**ID40**	0.006	-	0.004	0.003	-	-	-	-	0.002	0.000	-	-	-	0.003
**ID41**	-	-	0.005	-	-	-	-	-	-	0.000	-	-	-	0.003

Ancestors were evaluated for their differential marginal contributions to the affected and control cohorts. For both affected and control groups, ancestors with an average contribution of 0.5% or greater in either group, and that contributed to two or more non-overlapping cohorts, were analyzed. This resulted in the identification of a total of 41 individual ancestors for comparison. The difference in their marginal genetic contributions to affected and control cohorts were tested using a two-tailed paired t-test. The ancestors’ marginal genetic contributions are shown in Tables 3 and 4 and the paired t-test results are presented in Table 5.

**Table 5:** Pairwise two-tailed t-tests for the 41 ancestors with the highest average marginal contributions to two or more non-overlapping cohorts.

Ancestor	DF	t	P	FDR	Group
ID1	5	5.0863	< 0.01	*	Affected
ID11	12	3.3826	< 0.01	*	Affected
ID4	1	151.7273	< 0.01	*	Affected
ID8	12	4.354	< 0.001	*	Affected
ID2	9	3.0727	< 0.02	*	Affected
ID15	11	3.1227	< 0.01	*	Affected
ID26	3	3.7823	< 0.05	#	Affected
ID32^B^	3	14.4752	< 0.001	*	Affected
ID33	4	3.0569	< 0.05	#	Affected
ID34^B^	4	3.3738	< 0.05	#	Affected
ID35	8	4.6647	< 0.002	*	Affected
ID7	12	-9.4784	< 0.001	*	Control
ID9	12	-7.3222	< 0.001	*	Control
ID12	12	-7.3755	< 0.001	*	Control
ID17	12	-7.2343	< 0.001	*	Control
ID19	12	-6.4782	< 0.001	*	Control
ID20	12	-4.1063	< 0.002	*	Control
ID23	12	-6.0075	< 0.001	*	Control
ID28	12	-7.024	< 0.001	*	Control
ID29	12	-7.1564	< 0.001	*	Control
ID30	12	-4.2141	< 0.002	*	Control
ID38	10	-4.2976	< 0.002	*	Control
ID41^B^	12	-17.475	< 0.001	*	Control
ID37	12	-7.0793	< 0.001	*	Control
ID40^B^	12	-11.2122	< 0.001	*	Control
ID39^B^	12	-17.6036	< 0.001	*	Control
ID14	5	1.9418	N.S.		-
ID21	12	-1.209	N.S.		-
ID6	12	-1.372	N.S.		-
ID10	12	-1.9819	N.S.		-
ID13^B^	12	-1.3826	N.S.		-
ID3	4	2.5778	N.S.		-
ID16	12	1.3197	N.S.		-
ID18	12	-1.6611	N.S.		-
ID5	4	2.4813	N.S.		-
ID22	2	1.2627	N.S.		-
ID24	4	2.5977	N.S.		-
ID25	2	3.0586	N.S.		-
ID27^B^	12	0.5217	N.S.		-
ID31^B^	12	-0.3303	N.S.		-
ID36^B^	12	1.7789	N.S.		-

N.S.: contributions are not significantly different to affected or control cohorts;

^B^: denotes broodmares.

FDR: significant at an experimental-wise 5% (*) or 10% (#) false discovery rate.

The paired t-test revealed 26 significant ancestors, 11 significant to affected cohorts, and 15 significant to control cohorts. Of the latter, 12 were stallions and three were broodmares. Of the 11 ancestors significant to affected cohorts, nine were stallions, and two were broodmares. Of these 26 ancestors, 23 remained significant at an experimental-wise 5% FDR, while the remaining 3 ancestors were significant at a 10% FDR. S1 and S2 Tables provide additional information on significant stallions and broodmares (26 ancestors), including year of birth and gait. Statistical results for all 41 ancestors tested are shown in Table 5.

**Fig 2:**
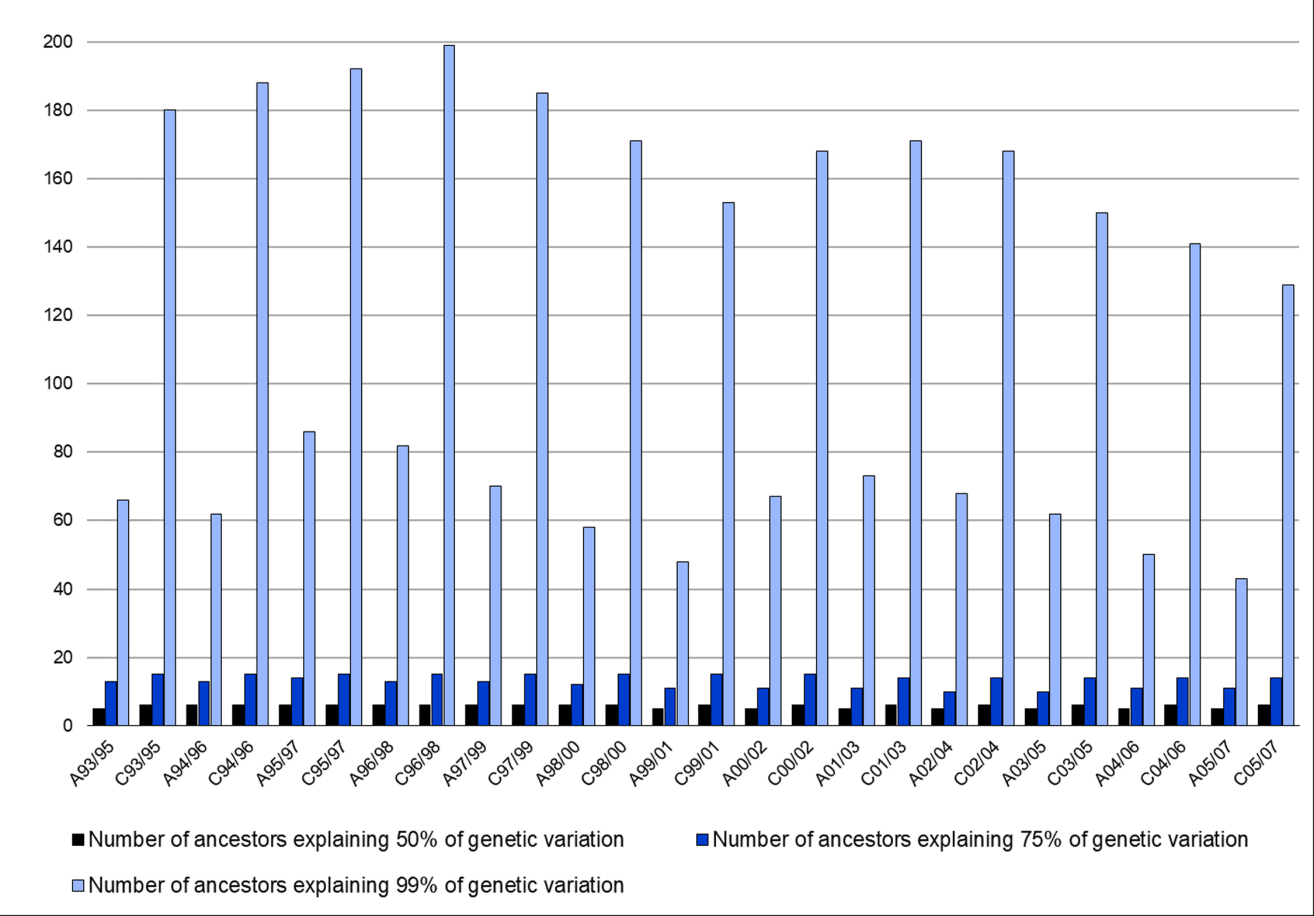
Number of ancestors explaining 50, 75, and 99% of the gene pool seen in 26 cohorts of control (C) or affected (A) racehorses.

For each control cohort, the number of ancestors necessary to explain 50% of the gene pool was 6. The same was true for 5 of the affected cohorts. The remaining 8, A93/95, A99/01, A00/02, A01/03, A02/04, A03/05, A04/06, and A05/07, needed only 5 ancestors to explain 50% of the gene pool. The fewest number of ancestors to explain 75% of the gene pool for a control cohort was 14, from C03/05 to C05/07. Only 10 ancestors were necessary to reach 75% of the gene pool in cohorts A02/04 and A03/05. Due to the presence of unknown ancestors in some of the pedigrees (the Std studbook was not closed to outside horses until 1973), it may not be possible to explain 100% of genetic pool. Therefore, the number of ancestors necessary to explain 99% of genetic variability was obtained. As few as 43 ancestors accounted for 99% of genetic variability in the A05/07 group, while 129 was the minimum required for C05/07. This difference may be due to the smaller number of individuals in the affected cohorts. Eighty-two ancestors explain almost the entire variability for A96/98, and 199 for C96/98, which are the largest cohorts. S3 Table presents complete details regarding the number of ancestors explaining 50%, 75%, and 99% of the genetics of each of the 26 affected and control cohorts, and the greatest marginal ancestral contribution to each. A pictorial representation of these results is shown in Fig 2.

**Fig 3:**
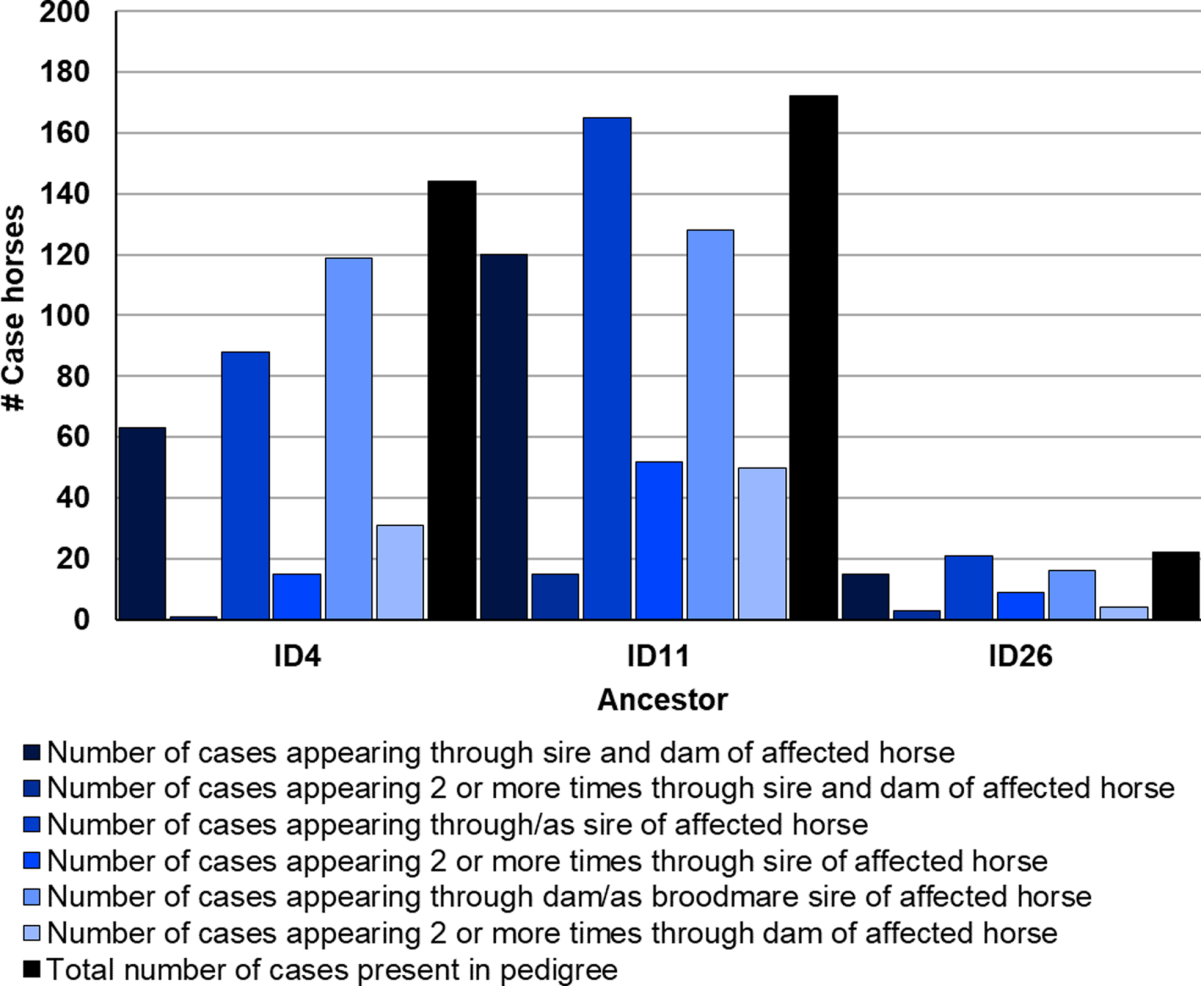
Number of five-generation pedigrees of affected Standardbred racehorses in which three significant ancestors appear once or multiple times and through the sire or dam of the horse, or through both.

**Fig 4:**
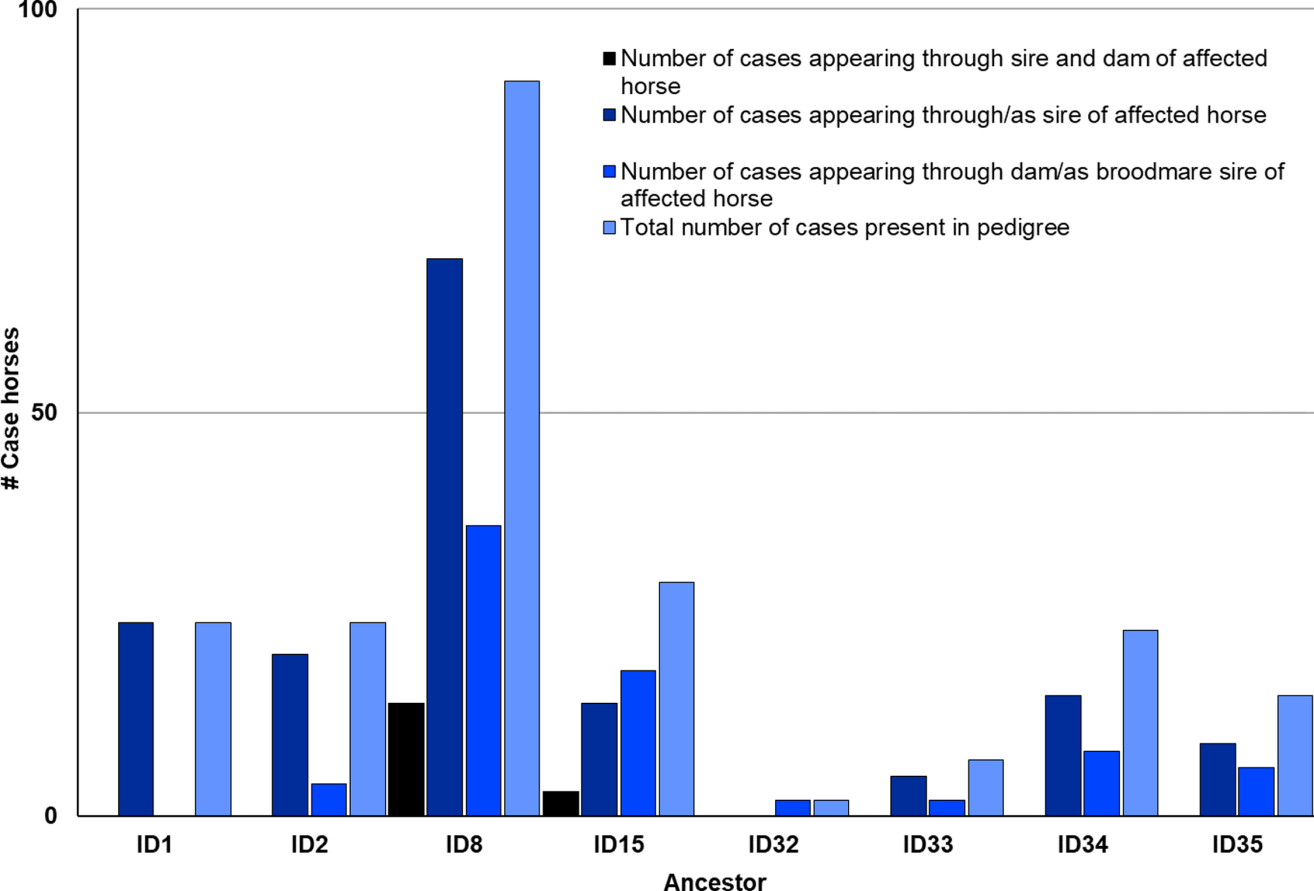
Number of five-generation pedigrees of affected Standardbred racehorses in which the remaining eight significant ancestors appear once through the sire or dam of the horse, or through both.

**Fig 5:**
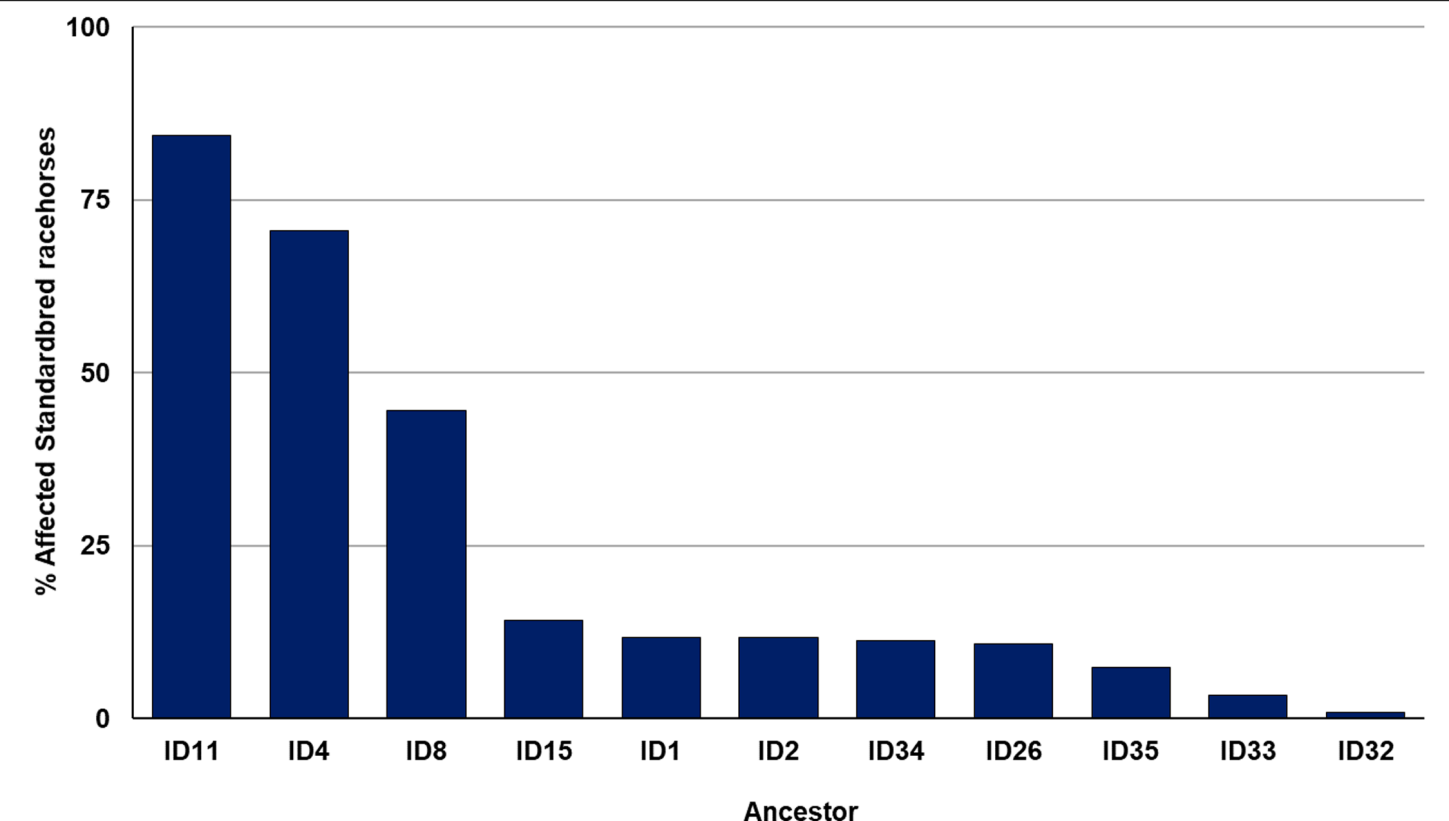
Percentage of affected horses which have each of 11 significant ancestors present at least once in their five-generation pedigree.

**Fig 6:**
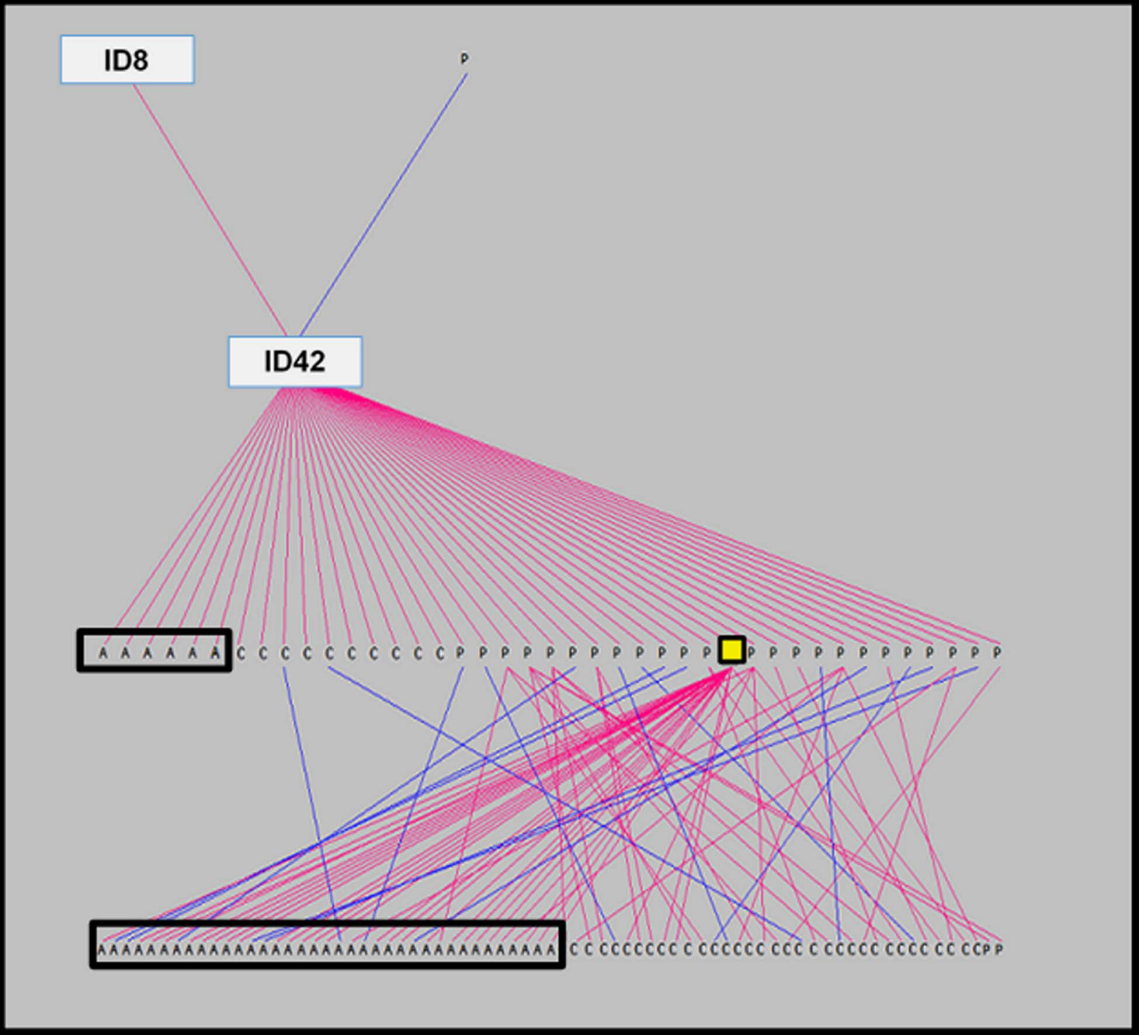
Sire line pedigree of ID1 (yellow square) depicting relatives, ancestors and descendants appearing as affected (A) or control (C) racehorses, or as ancestors in the pedigrees of other affected or control horses (P). Sire lines are presented in pink, with dam lines in blue.

After determining the ancestors that were significant to affected cohorts, the frequency of their appearance in the five-generation pedigrees of affected Std was examined. A number of affected Std had stallions ID4, ID11, or ID15 appear through both their sire and dam. These three particular ancestors appear at least once in 84.3%, 70.6%, and 10.8% of all cases of AF at OVCTH, respectively. Further data on ID4, ID11, and ID26 are shown in Fig 3. Of the remaining ancestors significant to affected cohorts, none appear through the sire or dam of affected horses more than once. However, ID8 appears in the five-generation pedigree of 44.6% of disease cases, whereas ID1 appears only as a sire of case horses. Fig 4 shows data for the remaining 8 significant ancestors, and the number of times each appears through the sire or dam of a case horse. Interestingly, ID4 and ID26 appear more frequently through the dam of affected racehorses than through their sire. Percentage of five-generation pedigrees of case horses in which a significant ancestor is present is shown in Fig 5. Lastly, after observing the regularity with which individuals from a particular sire line (ID1, ID8, ID42) were appearing in the case group, a pedigree diagram for this family, Fig 6, was constructed using PedigreeViewer [16].

**Fig 7:**
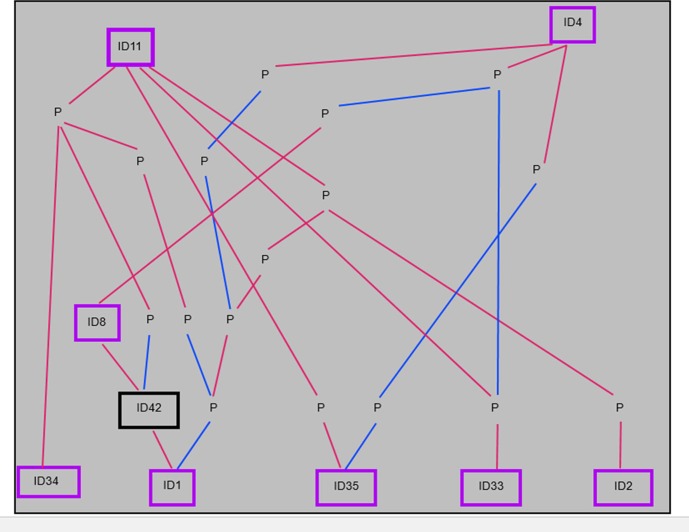
Diagram depicting the inter-relationships between eight of 11 ancestors significant to affected cohorts (outlined in purple) and one ancestor with affected offspring that is also a sire and broodmare sire of additional affected offspring (outlined in black), with ancestors in the pathways indicated by P. Sire lines are presented in pink, with dam lines in blue.

In this study, the majority of the stallions that contributed significantly to affected cohorts are from pacing bloodlines, but ID26 is from trotting bloodlines. Interestingly, many of the sires from pacing lineage are related to each other within fewer than five generations. ID1, ID2, ID33, ID34, and ID35 all descend from ID11. Additionally, ID1, ID8, ID33, and ID35 descend from ID4. Inter-relationships between these ancestors are shown in Fig 7. The sires which contributed more significantly to controls than to affected horses tend to appear more frequently as great-grandparents, or even further back in the pedigrees of affected and control horses. They are representative of both trotting and pacing lineages. The broodmare ID32 has two affected offspring, one of which is sired by ID33, himself significant to affected cohorts. Additionally, broodmare ID34 is significant, and is the dam of the stallion ID3, noted in Kraus et al. [7] for his large number of affected progeny.

## Discussion

An important information for managing genetic diversity of a population is the ancestors’ marginal contributions. For increasing or maintaining diversity, descendants of highly contributing ancestors might be used less for breeding and descendants from less contributing ancestors could be used more intensively. The same strategy might be used in the Std horse population, but aiming to manage AF incidence. The Pedig program developed by Boichard [14] has been employed by researchers around the globe, studying a variety of species. Italian, Colombian, and Irish cattle have been investigated by Bozzi *et al*. [17], Martínez *et al*. [18], and McParland *et al*. [19], respectively; and seven rare breeds of French sheep were studied by Danchin-Burge *et al*. [20]. A small number of studies have been conducted on the genetic variability of certain breeds of horse. Olsen *et al*. [21] studied the population structure of two Norwegian breeds, while the Dutch harness horse population was the topic of research by Schurink *et al*. [22]. Moreover, Pjontek *et al*. [23] investigated four Slovak breeds: the Hucul, Lipizzan, Shagya Arab, and Slovak Sport Pony. It is common for researchers using this software to compose reference populations according to year of birth, with cohorts covering from as few as one year [19], to as many as nine [21].

In horses, the largest marginal genetic contribution by a single ancestor tends to be higher than the values seen in other species. Valera *et al*. [24] reported 0.158 as the largest marginal contribution to an ancestor in the Andalusian, while Cervantes *et al*. [25] found two very highly contributing ancestors, at 0.168 and 0.163, in the Spanish Arabian horse. Two of the highest marginal contributions seen by a single ancestor were 0.229 and 0.193 by a pair of stallions in the Dutch harness horse stud book [22], eclipsed only by the contributions of 0.217 to the Døle and 0.261 to the Nordland/Lyngen, two Norwegian breeds, which were reported by Olsen *et al*. [21]. These reports on large marginal contributions by single ancestors validate our findings, which showed large marginal contributions to affected racehorses.

A common practice for applying marginal ancestral contributions to the genetic diversity of a population is to determine the number of ancestors required to explain differing percentages of the genetic pool. Only three ancestors of the Nordland/Lyngen breed explain 50% of the population’s genetic pool, while five account for the same variation in the Døle [21]. From three to 11 ancestors are necessary to explain 50% of the pool of four Slovak breeds, with as few as 17 to 82 ancestors contributing to the entire population of each [23]. The most similar population to that of this study, however, is once again that of the Dutch harness horse [22]. There, in the most recent cohort studied, four ancestors explain half of the population’s genetic pool, which is not surprising considering the extremely high contributions of the two ancestors mentioned above. An additional 10 ancestors were required to explain a further 25% of variation. Schurink *et al*. [22] stated that the two most influential ancestors are stallions, one born in 1950, and the other in 1975. In the current study the two most contributing ancestors to the analyzed cohorts were also stallions born much earlier in 1895 and 1926, respectively. Schurink *et al*. [22] reported similar numbers of ancestors accounting for 50% and 75% of the genetic pool as those found in the present study. Therefore, while the cohorts used in the present study are relatively small, the largest single marginal contribution by any single ancestor, and number of ancestors necessary to explain 50% and 75% of the gene pool, are comparable to those of previous studies on different breeds of horse. It is worth noting that inbreeding is not expected to cause bias in the results presented here, as a previous study [8] using a similar dataset examined the possible relationship between inbreeding and AF and no significant association was found.

The most interesting facet to the results of this study is the relatedness of sires that contribute significantly more to affected cohorts than controls. As clearly demonstrated in Fig 6, one particular line of sires accounts for a number of affected horses, which supports the finding through CFC [12] that affected horses have a greater degree of relatedness than controls. Within 3 ancestral generations of this sire line are 2 of the most significant sires of affected horses, ID1 and ID8. It can be seen that the sire of ID1 (ID42) is himself a sire of six affected horses. In addition, ID42 is the sire of three additional sires, and eight dams, of affected horses, with two sons in addition to ID1 shown to have sired more than one case horse themselves. As mentioned previously, ID1 descends from ID11, as do ID35, ID2, and ID33. This is very strong evidence for a genetic background underlying AF in the Std, particularly when taking into consideration the moderate heritability as described by Kraus et al. [7].

Stallion ID11 is one of the few ancestors significant to affected cohorts that also has consistent marginal genetic contributions to control cohorts, with an average marginal genetic contribution > 3%. This demonstrates the popularity of this particular ancestor not only as a stallion, but his continued influence on breeding practices today despite being born in the 1960s. It does seem from the results of the present study that a heavy dose of ID11 breeding in an individual could predispose a Std to AF (see Fig 3); however, not enough is known about the mode of inheritance of the arrhythmia to clearly show that this particular stallion is at fault. The results herein are simply a starting point, providing researchers with a definitive direction for future studies of this disease. It is possible that strategic breeding practices and avoiding this particular stallion’s bloodlines may help to decrease the incidence of the arrhythmia, but further investigation is required. One important direction of study, with the availability of the 60,000 SNP chip by Illumina [26], is conducting genome-wide association studies in Std, including this popular family. Results from that type of study would provide details on not only the potential causative mutation(s), but the mode of inheritance as well, and where the specific alleles associated with liability to the disease are coming from. Additionally, with the genotyping technology that is available today, there would be the potential to test and identify affected individuals at birth or a young age if a causative mutation was discovered.

## Conclusions

There is evidence that a number of stallions contribute significantly more highly to groups of Std affected with AF than to unaffected racing contemporaries. A number of these sires are related, being descendants of ancestors that contributed significantly more to groups of affected Std. The numerous appearances of many of these stallions in the five-generation pedigrees of affected horses demonstrate the current popularity of these bloodlines. The arrhythmia appears to be particularly prevalent in the descendants of one sire family. The close relationships of ancestors significant to affected Std indicates that there is a genetic background to AF in the breed. However, there was no indication of a simple mode of inheritance for AF in the Std.

Gathering more information on affected and healthy horses within these families, conducting genetic evaluations and genome-wide genotyping of horses, including influential sire lines, is the logical next step in determining the genetic background of AF in the Std and better understanding how to manage genetic liability to the arrhythmia.

## Supporting information

S1 Table Additional information on significant stallions. (DOCX)

S2 Table Additional information on significant broodmares. (DOCX)

S3 Table Number of ancestors contributing to 50%, 75% and 99% of genetic pool of each cohort, as well as the largest individual marginal contribution to each. (DOCX)
