# Supplementary material for: Marginal ancestral contributions to atrial fibrillation in the Standardbred racehorse: Comparison of cases and controls
Source: PLoS One. 2018 May 15;13(5):e0197137. doi: 10.1371/journal.pone.0197137 (PMC5953485; doi:10.1371/journal.pone.0197137)
Supplement: S1 Table — (DOCX) [file pone.0197137.s001.docx]

**S1 Table.** Additional information on significant stallions.

| **Sire** | **Year of birth** | **Gait** | **Significant to** | **Total number of progeny*** |
| --- | --- | --- | --- | --- |
| ID29 | 1903 | Both | Control | 41 |
| ID38 | 1944 | Pace | Control | 1,074 |
| ID4 | 1948 | Pace | Affected | 1,371 |
| ID11 | 1960 | Pace | Affected | 1,266 |
| ID15 | 1974 | Pace | Affected | 1,993 |
| ID8 | 1975 | Pace | Affected | 1,817 |
| ID33 | 1984 | Pace | Affected | 1,453 |
| ID2 | 1987 | Pace | Affected | 2,062 |
| ID35 | 1987 | Pace | Affected | 847 |
| ID1 | 1997 | Pace | Affected | 877 |
| ID7 | 1895 | Trot | Control | 393 |
| ID9 | 1902 | Trot | Control | 498 |
| ID17 | 1910 | Trot | Control | 226 |
| ID19 | 1925 | Trot | Control | 530 |
| ID37 | 1925 | Trot | Control | 279 |
| ID20 | 1936 | Trot | Control | 526 |
| ID23 | 1944 | Trot | Control | 506 |
| ID28 | 1947 | Trot | Control | 915 |
| ID26 | 1954 | Trot | Affected | 704 |
| ID30 | 1968 | Trot | Control | 2,209 |
| ID12 | 1849 | Unknown | Control | 89 |

- Total number of progeny registered with Standardbred Canada as of 2010.

** Ancestor not significant in the statistical analysis.
