# Supplementary material for: Marginal ancestral contributions to atrial fibrillation in the Standardbred racehorse: Comparison of cases and controls
Source: PLoS One. 2018 May 15;13(5):e0197137. doi: 10.1371/journal.pone.0197137 (PMC5953485; doi:10.1371/journal.pone.0197137)
Supplement: S2 Table — (DOCX) [file pone.0197137.s002.docx]

**S2 Table.** Additional information on significant broodmares.

| Broodmare | Year of birth | Gait | Significant to | Total number of progeny* |
| --- | --- | --- | --- | --- |
| ID34 | 1979 | Pace | Affected | 17 |
| ID32 | 1983 | Pace | Affected | 5 |
| ID40 | 1906 | Unknown | Control | 14 |
| ID41 | 1917 | Trot | Control | 3 |
| ID39 | 1925 | Trot | Control | 13 |

* Total number of progeny registered with Standardbred Canada as of 2010.
