# Supplementary material for: Marginal ancestral contributions to atrial fibrillation in the Standardbred racehorse: Comparison of cases and controls
Source: PLoS One. 2018 May 15;13(5):e0197137. doi: 10.1371/journal.pone.0197137 (PMC5953485; doi:10.1371/journal.pone.0197137)
Supplement: S3 Table — (DOCX) [file pone.0197137.s003.docx]

| **S3 Table.** Number of ancestors contributing to 50%, 75% and 99% of genetic pool of each cohort, as well as the largest individual marginal contribution to each. | | | | |
| --- | --- | --- | --- | --- |
| **Cohort** | **Largest marginal contribution** | **50%*** | **75%*** | **99%*** |
| **A93/95** | 0.1265 | 5 | 13 | 66 |
| **C93/95** | 0.1250 | 6 | 15 | 180 |
| **A94/96** | 0.1281 | 6 | 13 | 62 |
| **C94/96** | 0.1213 | 6 | 15 | 188 |
| **A95/97** | 0.1418 | 6 | 14 | 86 |
| **C95/97** | 0.1207 | 6 | 15 | 192 |
| **A96/98** | 0.1466 | 6 | 13 | 82 |
| **C96/98** | 0.1224 | 6 | 15 | 199 |
| **A97/99** | 0.1437 | 6 | 13 | 70 |
| **C97/99** | 0.1200 | 6 | 15 | 185 |
| **A98/00** | 0.1409 | 6 | 12 | 58 |
| **C98/00** | 0.1211 | 6 | 15 | 171 |
| **A99/01** | 0.1413 | 5 | 11 | 48 |
| **C99/01** | 0.1203 | 6 | 15 | 153 |
| **A00/02** | 0.1420 | 5 | 11 | 67 |
| **C00/02** | 0.1186 | 6 | 15 | 168 |
| **A01/03** | 0.1739 | 5 | 11 | 73 |
| **C01/03** | 0.1210 | 6 | 14 | 171 |
| **A02/04** | 0.2000 | 5 | 10 | 68 |
| **C02/04** | 0.1221 | 6 | 14 | 168 |
| **A03/05** | 0.2051 | 5 | 10 | 62 |
| **C03/05** | 0.1247 | 6 | 14 | 150 |
| **A04/06** | 0.1481 | 5 | 11 | 50 |
| **C04/06** | 0.1255 | 6 | 14 | 141 |
| **A05/07** | 0.1579 | 5 | 11 | 43 |
| **C05/07** | 0.1272 | 6 | 14 | 129 |
| * Number of ancestors necessary to explain this proportion of the genetic pool. | | | | |
